# Patient hematology during hospitalization for viral pneumonia caused by SARS-CoV-2 and non-SARS-CoV-2 agents: a retrospective study

**DOI:** 10.1186/s40001-021-00515-9

**Published:** 2021-05-14

**Authors:** Bingke Bai, Zhe Xu, Yan Hu, Mengmeng Qu, Juan Cheng, Shengdong Luo, Zengtao Yao, Hongyan Gao, Yenv Ma, Rong Gao, Jun Hou, Shaojie Xin, Panyong Mao

**Affiliations:** 1 Research Center of Clinical and Translational Medicine, Fifth Medical Center of Chinese, PLA General Hospital, 100 Middle Street of 4th West Ring Road, Beijing, 100039 China; 2 Treatment and Research Center for Infectious Diseases, Fifth Medical Center of Chinese, PLA General Hospital, 100 Middle Street of 4th West Ring Road, Beijing, 100039 China; 3 Liver Failure Treatment and Research Center, Fifth Medical Center of Chinese, PLA General Hospital, 100 Middle Street of 4th West Ring Road, Beijing, 100039 China

**Keywords:** Hematology, SARS-CoV-2, COVID-19, Adenovirus, H1N1

## Abstract

**Background:**

Hematological comparison of coronavirus disease (COVID-19) and other viral pneumonias can provide insights into COVID-19 treatment.

**Methods:**

In this retrospective case–control single-center study, we compared the data of 126 patients with viral pneumonia during different outbreaks [severe acute respiratory syndrome (SARS) in 2003, influenza A (H1N1) in 2009, human adenovirus type 7 in 2018, and COVID-19 in 2020].

**Results:**

One of the COVID-19 characteristics was a continuous decline in the hemoglobin level. The neutrophil count was related to the aggravation of COVID-19 and SARS. Thrombocytopenia occurred in patients with SARS and severe COVID-19 even at the recovery stage. Lymphocytes were related to the entire course of adenovirus infection, recovery of COVID-19, and disease development of SARS.

**Conclusions:**

Dynamic changes in hematological counts could provide a reference for the pathogenesis and prognosis of pneumonia caused by respiratory viruses in clinics.

## Background

Coronavirus disease (COVID-19) caused by a novel coronavirus, severe acute respiratory syndrome coronavirus 2 (SARS-CoV-2), emerged in China and has rapidly spread worldwide. As of March 3, 2021, more than 114.79 million confirmed cases and 2.5 million deaths (2.22% case fatality) in more than 200 countries have been reported [1]. Fever, fatigue and dry cough are considered the main clinical manifestations. In severe cases, dyspnea and/or hypoxemia may occur after 1 week of onset and, in extreme cases, can degenerate to acute respiratory distress syndrome, septic shock, metabolic acidosis, hemorrhage and coagulation dysfunction, multiple organ failure and death [2–4]. Although SARS-CoV-2 is a coronavirus similar to SARS-CoV and Middle East respiratory syndrome (MERS)-CoV, a higher human-to-human transmission rate has been observed in the COVID-19 pandemic than in the SARS and MERS outbreaks, which had shorter incubation periods and higher fractions of severe cases and deaths. Until recently, several studies have revealed that some specific minor symptoms are also correlated with COVID-19, such as anosmia, ageusia, and cutaneous manifestation [5–9].

Respiratory viruses infect and affect the upper and lower respiratory tract, respectively, in vulnerable populations, such as infants, the elderly, and immune-compromised individuals. Therefore, they could lead to pneumonia and various respiratory distress syndromes [10, 11]. These most probably result from coronavirus infections, such as MERS-CoV, SARS-CoV, and SARS-CoV-2, and other RNA viruses, such as influenza (H1N1, H5N1) and Ebola viruses, which have animal reservoirs and can cross species barriers to adapt to new environments and/or new hosts. These zoonoses may have disastrous consequences in humans [12]; the burden is even higher when they have neurological consequences. The most severe H1N1 influenza pandemic occurred in 1918, claiming over 50 million lives [13], while the last H1N1 pandemic in 2009 claimed approximately 200,000 lives worldwide [14]. Human adenoviruses [AdV], including type 55 and type 7, have been reported in the United States [15], Turkey [16], Singapore [17], and China [18].

The Chinese government applied the experience of SARS outbreak management toward controlling the COVID-19 epidemic. To date, the treatments adopted were based on previous experience with SARS, MERS, or influenza. Hematological findings are important in providing clinical teams with data on useful prognostic markers. Hematological changes in patients with COVID-19 and SARS are common. These patients usually have higher leukocyte counts and neutrophil-lymphocyte ratios, and lower lymphocyte counts and monocyte, eosinophil, and basophil percentages, especially in severe cases. Most severe cases demonstrated elevated levels of infection-related biomarkers and inflammatory cytokines [19–21]. Another laboratory finding that can be easily measured in daily clinical practice is albumin, whose concentration levels may be associated with COVID-19 severity [22]. In 45 patients infected with AdV, leukocytosis with left shift was noted in the early course; leukopenia and thrombocytopenia were found as disease severity progressed [23]. Investigations of leukocyte and platelet (PLT) counts in the peripheral blood are useful as they may constitute ancillary exams to form assumptions.

Paliogiannis et al. [24] published their study focusing on COVID-19 and COVID-19 pneumonia differences. Although the clinical characteristics are known, studies on the kinetics of hematological changes in patients with COVID-19 are scarce. Interestingly, most studies have focused on differences and similarities between SARS-CoV-2 and other beta-coronaviruses (primarily SARS-CoV and MERS-CoV), but few of them have examined other viruses, such as influenza viruses (primarily H1N1, but also H5N1 and H7N9) and adenoviruses [25, 26]. Recent research has mostly focused on admission indicators, with few studies on changes in lymphocytes throughout the disease course. Zheng et al. [20] compared the differences in patients with COVID-19 to reveal the risk factors for severe disease. Different patients may have a different disease course (disease development or recovery period) after the disease onset, which corresponds to different changes in the white blood cell (WBC) counts. The accuracy of the analysis results for dynamic changes would be affected by using the days after disease onset as the only reference; however, this ignores the different disease course in different patients.

The COVID-19 pandemic continues, and virus transmission may last for more than 1 year [27]. Concurrently, other coronaviruses and influenza viruses may co-infect people with SARS-CoV-2 and cause more serious respiratory diseases, owing to similar clinical symptoms [28]. Whether SARS-CoV-2 or other respiratory viruses can promote each other’s spread remains unclear. Early diagnosis and treatment are very important for the prevention and control of infectious diseases. This retrospective, single-center study aimed to analyze the dynamic changes in hematological counts in four different patients with viral pneumonia at our hospital during several pneumonia outbreaks (SARS, 2003; H1N1, 2009; human AdV type 7, 2018; COVID-19, 2020) and provide reference values for the pathogenesis and prognosis of pneumonia caused by respiratory viruses.

## Methods

### Study population

For this retrospective, single-center case–control study, we recruited 49 patients with COVID-19 pneumonia diagnosed according to World Health Organization (WHO) guidelines (Diagnostic testing for SARS-CoV-2, https://www.who.int/emergencies/diseases/novel-coronavirus-2019/technical-guidance-publications). We examined the data of 77 hospitalized patients with pneumonia infected with SARS-CoV (year: 2003; *n* = 34), AdV 7 (year: 2018; *n* = 21), or H1N1 (year: 2009; *n* = 22), whose clinical information preserved in our hospital biobank. The study was approved by the Medical Ethical Committee of our hospital (the approval number: 2020066D). All patients provided written informed consent to participate. We also collected the clinical information of 90 people from the healthy cohort in our biobank. All enrolled patients had fully recovered and were discharged alive.

### Data collection

We reviewed the clinical charts, nursing cards, and laboratory findings of all patients. Data on general information, such as sex, age, routine blood tests, treatment, and outcomes, were collected from medical records. Clinical outcomes were followed up until May 5, 2020. Routine blood tests were conducted, including the assessment of the WBC, neutrophil, lymphocyte, monocyte, eosinophils, basophil, hemoglobin (HGB), and PLT levels during hospitalization. We collected information on only WBC, neutrophils, lymphocytes, and PLT levels of patients with SARS because the blood cell analyzer used was not advanced at the time of diagnosis. The patients’ age at hospitalization was recorded. Severe and mild disease classifications were based on clinical guidelines. Soon after onset, we collected data from the test performed closest to the onset date (i.e., within 3 days after hospitalization; early stage); for information on the complete recovery period, we collected the final data from the final test (i.e., within 2 days before discharge) to assess the disease stage. Then, the disease course was divided into three equal periods: the initial, obviously symptomatic, and recovery stages. The subtype definition of patients with COVID-19 was based on the diagnosis and treatment scheme for SARS-CoV-2 (5th edition) applied in China with minor modifications of the WHO standards (Diagnostic testing for SARS-CoV-2, https://www.who.int/emergencies/diseases/novel-coronavirus-2019/technical-guidance-publications). Specifically, the severity of COVID-19 was categorized as mild, common, severe, or critical. For better analysis, we grouped the patients as mild (including mild and common) and severe (including severe and critical).

### Statistical analysis

Statistical analyses were performed using SPSS software (version 21.0; IBM Corp., Armonk, NY, USA). Quantitative variables were tested for normality of distribution. Medians and interquartile ranges were calculated and analyzed using non-parametric tests. Particularly, the Mann–Whitney *U* and Kruskal–Wallis *H* tests were used to analyze the values between two and three or more groups, respectively. The figures were drawn using GraphPad Prism 8 (San Diego, CA, USA). *P* values < 0.05 were considered significant.

## Results

### Demographic and epidemiologic characteristics

The study population comprised 126 hospitalized patients, including 49, 34, 21 and 22 patients with COVID-19, SARS, AdV pneumonia, and pneumonia caused by H1N1, respectively. Additionally, the data of 90 healthy people were examined. We categorized the patients into the COVID-19, SARS, AdV and H1N1 groups, respectively.

In the COVID-19 group, the median patient age was 47.4 years (range 13–85 years); 27 of 49 patients (55.1%) were male, 25 (51.0%) had traveled or lived in Wuhan, and 19 (38.8%) were in close contact with COVID-19 patients.

In the SARS group, the median patient age was 29.1 years (range 13–54 years), and 13 of 34 in this group (38.2%) were male.

The mean patient age in the AdV group was 20.7 years (range 18–26 years). The AdV epidemic was an outbreak in a dense and closed environment, and the workers were all young men.

In the H1N1 group, the median patient age was 21.7 years (range 23–85 years), and 18 of 22 patients in this group (81.8%) were male.

### Hematological parameters in the COVID-19 group

**Fig. 1:**
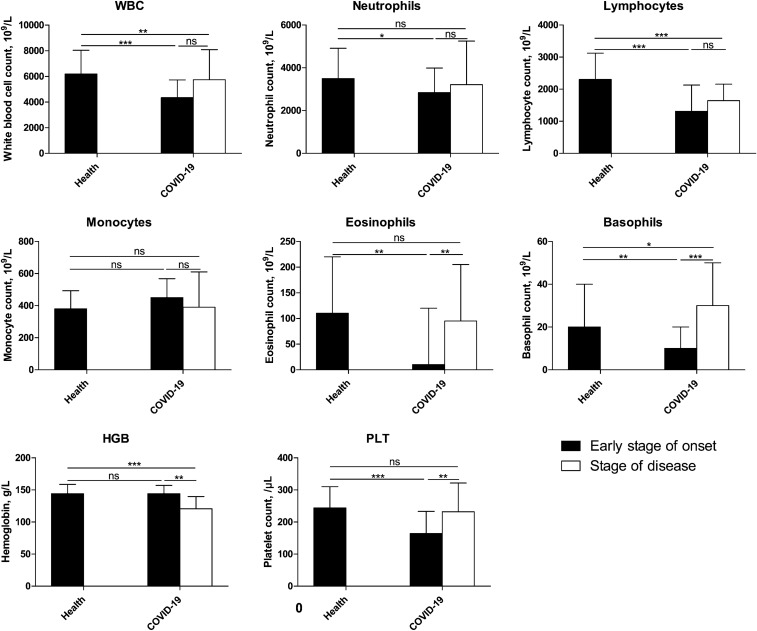
Comparison of COVID-19 blood test results between the early stage of onset and the stage of disease. The blood test results were collected from patients with COVID-19, and the data of the early stage of onset were obtained after performing the test closest to the onset date (i.e., within 3 days after hospitalization); information on stage of disease was obtained on the final test (i.e., within 2 days before discharge). The data were analyzed using SPSS software version 21. *P* values between 0.01 and 0.05, 0.001 and 0.01, and 0.0001–0.001 were considered statistically significant (*), very significant (**), and extremely significant (***), respectively. *P* values > 0.01 were considered not significant

First, we compared the early stage of onset and the stage of disease in the COVID-19 group. As shown in Fig. 1, the number of WBCs, neutrophils, lymphocytes, eosinophils, and PLTs decreased in the early stage of onset, with a significant decrease in the lymphocyte and PLT counts (*P* = 0.000). The HGB and monocyte levels remained unchanged (*P* > 0.05). The stage of disease showed reduced monocytes and HGB levels and elevations in the WBC, neutrophils, lymphocytes eosinophil, basophil, and PLT levels compared with the early stage of onset; however, only the differences in eosinophils, basophils, HGB, and PLTs were significant (*P* < 0.01).

**Fig. 2:**
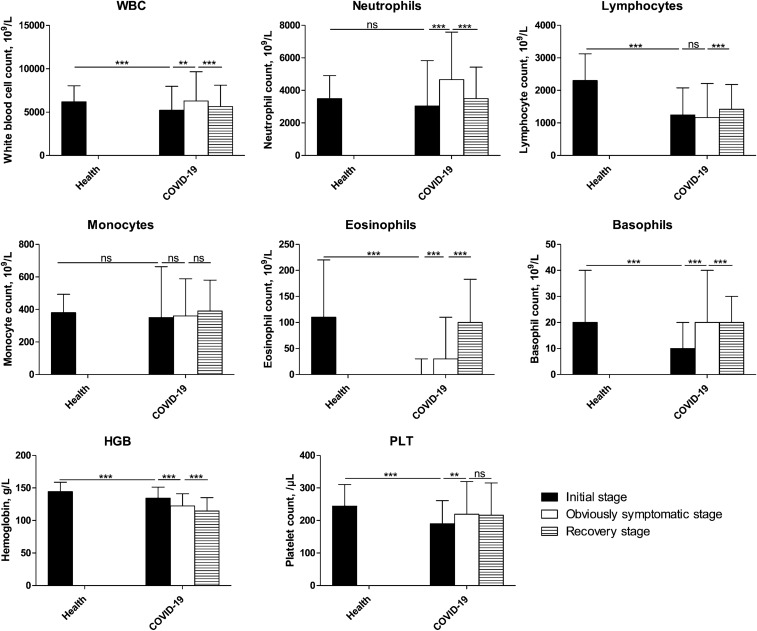
Characterization of the blood test results during the COVID-19 course. The blood test results were collected from patients with COVID-19, and the data of the early stage of onset were obtained after performing the test closest to the onset date (i.e., within 3 days after hospitalization); information on stage of disease was obtained on the final test (i.e., within 2 days before discharge). Then, the disease course was divided into three equal periods: the initial, obviously symptomatic, and recovery stages. The data were analyzed using SPSS software version 21. *P* values between 0.01 and 0.05, 0.001 and 0.01, and 0.0001–0.001 were considered statistically significant (*), very significant (**), and extremely significant (***), respectively. *P* values > 0.01 were considered not significant

During the disease course (Fig. 2), at the prodromal stage, the blood cell counts declined, and all reductions were significant (*P* = 0.000), except for neutrophil and monocyte counts (*P* > 0.05). The obviously symptomatic stage showed higher counts than the prodromal stage, apart from the lymphocyte and monocyte counts, which remained unchanged, and the HGB levels, which continuously decreased from the early stage of onset. The WBC, neutrophil, and HGB levels decreased significantly at the recovery stage (*P* < 0001), while the lymphocyte and eosinophil levels significantly increased (*P* < 0.001). Monocytes showed no significant changes throughout the disease course. With disease progression, the WBC counts increased, mainly because of the increased neutrophil count.

**Fig. 3:**
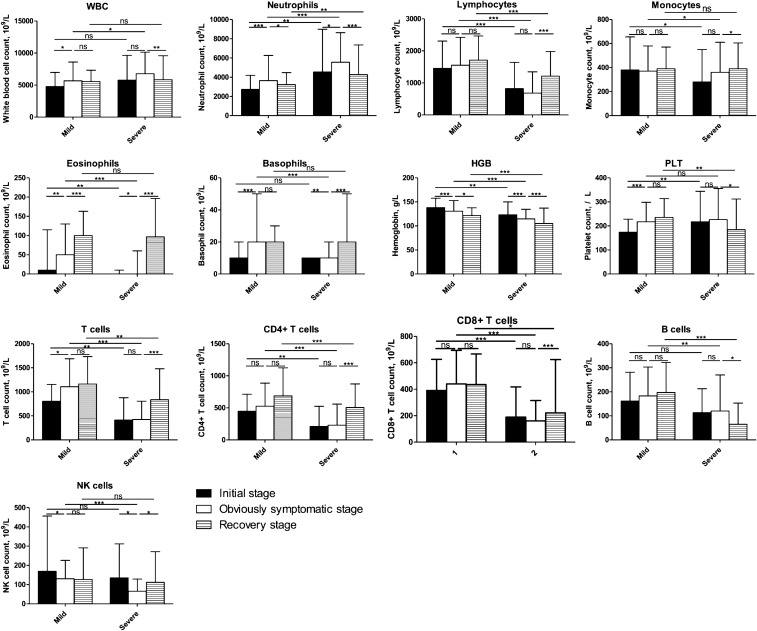
Comparison of the blood test results between patients with severe and mild COVID-19. The blood test results were collected from patients with COVID-19, and the data of the early stage of onset were obtained after performing the test closest to the onset date (i.e., within 3 days after hospitalization); information on stage of disease was obtained on the final test (i.e., within 2 days before discharge). Then, the disease course was divided into three equal periods: the initial, obviously symptomatic, and recovery stages. The data were analyzed using SPSS software version 21. *P* values between 0.01 and 0.05, 0.001 and 0.01, and 0.0001–0.001 were considered statistically significant (*), very significant (**), and extremely significant (***), respectively. *P* values > 0.01 were considered not significant

Further analysis of the difference between patients with mild and severe COVID-19 (Fig. 3) showed that the WBC count in those with severe disease was significantly higher than the corresponding count in those with mild disease during the obviously symptomatic stage (*P* = 0.011). Throughout the entire disease course, the neutrophil count was consistently higher in severe COVID-19 patients compared to those with mild COVID-19 (*P* < 0.05), while the converse was true for the lymphocyte count (*P* = 0.000). The trend in the lymphocyte count in severe COVID-19 patients was consistent with that observed in the whole COVID-19 group, suggesting that the lymphocytes are related to disease severity. The HGB count was consistently lower in severe than in mild COVID-19 patients. A decline in the HGB count could lead to a decrease in the oxygen-carrying capacity and the oxygen levels in the blood; this reduces the oxygen saturation of patients, leading them to a sensation of hardly breathing. For severe disease, the monocyte count was consistently low and normalized during the recovery period, while for the mild disease, it remained unchanged throughout the disease course. The PLT count in patients with severe disease remained unchanged until the recovery stage; however, in patients with mild disease, it increased with disease progression and remained unchanged at the recovery stage.

Flow cytometry was used to verify the change in lymphocyte count. Analysis of lymphocytes indicated that the CD4  + and CD8  + T cell counts were always lower in severe than in mild patients, but the ratio of CD4  + to CD8  + T cells was not significantly different between them. The CD3  + T cell count increased in patients with mild COVID-19 before the obviously symptomatic stage, while in those with severe COVID-19, the count increased later. Increases in the CD3  + T cell count in patients were related to improvements in their conditions. There were no significant changes in B-cell counts, except for a decrease in severe patients with disease recovery (*P* = 0.015). At the obviously symptomatic and recovery stages, the B-cell count was significantly lower in patients with a severe than in those with a mild disease condition (*P* = 0.022 and *P* = 0.015, respectively), indicating that the humoral immune system may be impaired in severe patients. The prodromal stage showed a higher natural killer cell count than the obviously symptomatic stage in severe and mild patients; however, the recovery stage in severe patients showed a significant increase in this count (*P* = 0.026) and that in mild patients showed no significant change.

### Comparative analysis of hematological parameters in the COVID-19 and other viral pneumonia groups

**Fig. 4:**
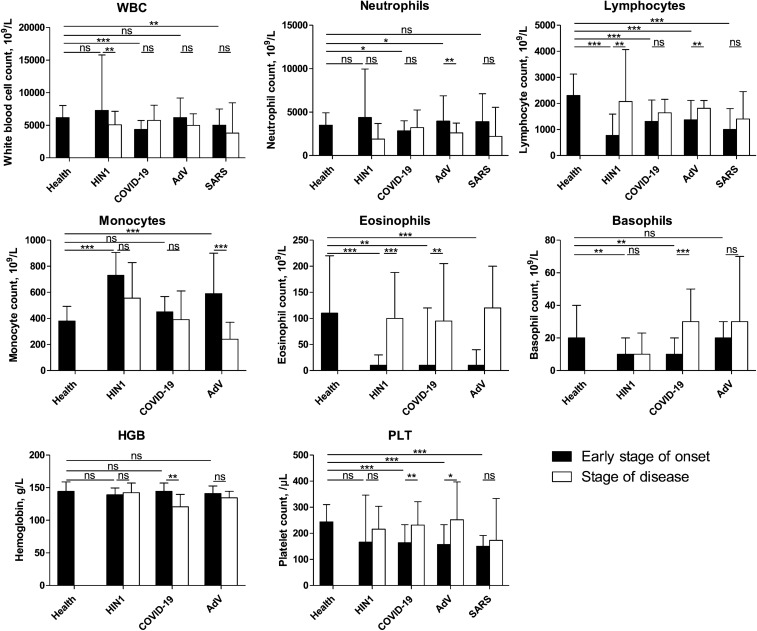
Blood tests results for each of the four groups between the early stage of onset and the disease stage. Blood test results were collected from patients with pneumonia secondary to COVID-19, SARS, AdV, or H1N1 during hospitalization. The data of the early stage of onset were obtained after performing the test closest to the onset date (i.e., within 3 days after hospitalization); information on stage of disease were obtained on the final test (i.e., within 2 days before discharge). Then, the disease course was divided into three equal periods: the initial, obviously symptomatic, and recovery stages. The data were analyzed using SPSS software version 21. *P* values between 0.01 and 0.05, 0.001 and 0.01, and 0.0001–0.001 were considered statistically significant (*), very significant (**), and extremely significant (***), respectively. *P* values > 0.01 were considered not significant

At the early stage of onset, all groups showed a decrease in lymphocyte and eosinophil counts and no significant change in HGB counts compared with healthy individuals (Fig. 4). Only the COVID-19 and SARS groups showed a decreased WBC count (*P* < 0.05), indicating that the two diseases may have similar pathogeneses. The AdV group showed a contrasting result to the COVID-19 group, as the neutrophil count increased (*P* = 0.026), while the SARS and H1N1 groups showed no significant changes (*P* > 0.05). Unlike the COVID-19 group, the monocyte count increased in the H1N1 and AdV groups (*P* = 0.000 and 0.019, respectively). Except for the H1N1 group, the PLT count decreased significantly in other groups soon after onset. When comparing the complete recovery period with the early onset stage, the WBC count was found to be low only in the H1N1 group (*P* = 0.007), and the neutrophil and monocyte counts decreased only in the AdV group (*P* = 0.009 and 0.000, respectively). Additionally, the lymphocyte count increased in the H1N1 and AdV groups (*P* = 0.002 and 0.005, respectively). The AdV group showed an increased PLT count (*P* = 0.012), similar to the COVID-19 group (*P* = 0.010). Only the COVID-19 group showed a decreased HGB count (*P* = 0.002), which was below the normal level. The WBC, lymphocyte, and neutrophil counts dropped below the normal level (*P* < 0.05) in all groups, except for the neutrophil count in the COVID-19 group (*P* = 0.354). The PLT count remained below the normal level during the complete recovery period only in the SARS group.

**Fig. 5:**
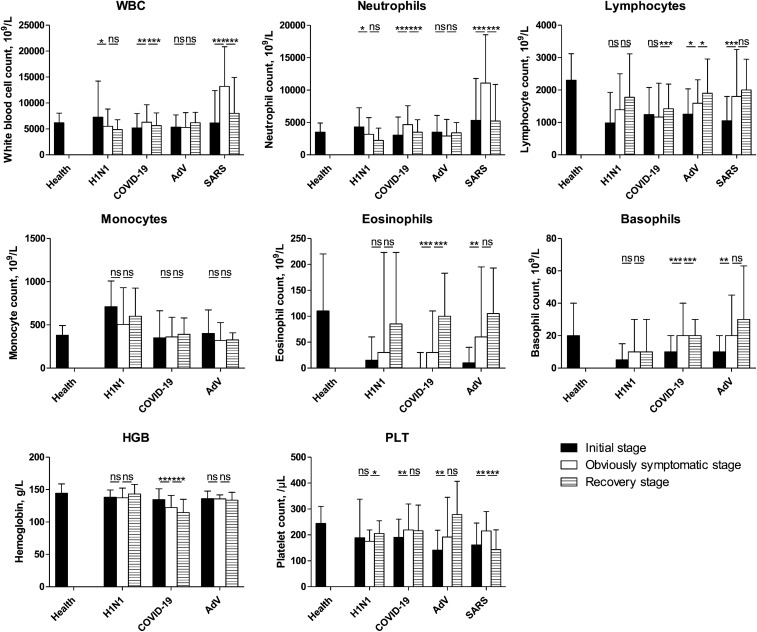
Characterization of the four groups of blood tests during the course of COVID-19. Blood test results were collected from patients with pneumonia secondary to COVID-19, SARS, AdV, or H1N1 during hospitalization. The data of the early stage of onset were obtained after performing the test closest to the onset date (i.e., within 3 days after hospitalization); information on stage of disease were obtained on the final test (i.e., within 2 days before discharge). Then, the disease course was divided into three equal periods: the initial, obviously symptomatic, and recovery stages. The data were analyzed using SPSS software version 21. *P* values between 0.01 and 0.05, 0.001 and 0.01, and 0.0001–0.001 were considered statistically significant (*), very significant (**), and extremely significant (***), respectively. *P* values > 0.01 were considered not significant

Regarding the WBC and neutrophil counts during the whole disease course (Fig. 5), the patients in the SARS group showed a similar trend to those in the COVID-19 group, especially those with severe COVID-19. Unlike the other three groups, the H1N1 group showed a decrease in the WBC and neutrophil counts during the disease development period. There were no significant changes in the WBC and neutrophil counts in the AdV group. An increase in the lymphocyte count was observed during the recovery period in the COVID-19 (especially in severe patients) and AdV groups and during the development period in the SARS group. The SARS and COVID-19 groups showed a significantly lower PLT count than that in healthy individuals. The SARS group showed an increasing trend in the PLT count during the disease development period, similar to mild patients in the COVID-19 group and those in the AdV group, and a decreasing trend during the recovery period, similar to that in the severe COVID-19 and H1N1 groups. Only the COVID-19 group showed a decreasing trend in the HGB count. The monocyte count did not significantly change during the disease course in all groups; however, the H1N1 group had a higher count compared to the healthy group, and the AdV group showed a lower count during the obviously symptomatic and recovery stages.

## Discussion

Consistent with previous works [3, 19, 20], the lymphocyte counts in all patients were lower than the normal level during the disease course. The lymphocyte count was lower in severe than in mild COVID-19 patients, possibly indicating that lymphocytopenia is related to disease severity. Lymphocytopenia has been evaluated as an independent biomarker of mortality in hospitalized patients diagnosed with community-acquired pneumonia [29] and is considered a risk factor for influenza during bacterial superinfections [30], thus determining a bad prognosis. Lymphopenia has also been associated with an increased risk of death in patients with COVID-19 [7]. Patients with lymphocytopenia would require close clinical monitoring for these potentially fatal infectious complications.

Neutrophilia and thrombocytopenia were observed in the severe COVID-19 and SARS groups. The neutrophil counts increased gradually in mild COVID-19, severe COVID-19, and SARS patients. The main neutrophil functions are phagocytosis, granules release, and cytokine production. Proinflammatory cytokines produced by neutrophils may limit virus replication and halt progression to severe disease. During disease recovery, the neutrophil counts gradually return to normal levels, implying that increasing and decreasing neutrophil counts may be signs of disease progression and recovery. Steroids may cause neutrophilia and have been used in the treatment of severe SARS and severe COVID-19. Neutrophils are reportedly susceptible to influenza A virus (IAV), and the infection is productive for pH1N1 [31]. Virus-infected neutrophils can upregulate interferons and other antiviral factors [32] that could limit viral replication. Neutropenia has been demonstrated to increase the pulmonary virus titer and the mortality rate during IAV infection, suggesting a protective role of neutrophils during influenza [33].

Upon antigen recognition, PLTs become activated and interact with WBC to facilitate pathogen clearance through WBC activation and clot formation [34]. The PLT counts of patients with AdV and mild COVID-19 were elevated at the obviously symptomatic stage and returned to normal levels at the recovery stage, while in the severe COVID-19 group, they decreased significantly after the obviously symptomatic stage. It has been reported that the most common hematological changes in patients with SARS and COVID-19 were lymphopenia and thrombocytopenia [20, 21]. Interestingly, these changes were closely related to the disease prognosis and reflected a more severe clinical course. A more sizable reduction in the PLT count was especially noted in non-survivors [35]. Viral infections elicit a systemic inflammatory response and cause an imbalance between procoagulant and anticoagulant homeostatic mechanisms [36]. Moreover, the development of autoimmune antibodies or immune complexes triggered by viral infection may contribute to thrombocytopenia development; SARS-CoV may also directly infect hematopoietic stem/progenitor cells, megakaryocytes, and PLT, inducing their growth inhibition and apoptosis [37]. Damaged lungs of patients with SARS may also induce thrombocytopenia [38]. The clinical value of a low PLT count has been assessed in a diagnostic model, which included thrombocytopenia (in addition to myalgia, fever, diarrhea, rhinorrhea, sore throat and lymphopenia) and effectively detected SARS-CoV-1 with 100% sensitivity and 86.3% specificity [39]. Elevated PLT counts at the obviously symptomatic stage in the mild COVID-19, AdV and SARS groups suggested that the PLT levels change with disease development and are related to thrombosis. Decreases in the PLT count, which were observed in the recovery period in the severe COVID-19 and SARS groups, indicated a hemorrhage risk of the disease. Patients with mild COVID-19 and AdV pneumonia may have better clinical outcomes compared to those with other pneumonias.

The monocyte count was increased during the course of H1N1 disease in our study. Monocytes are recruited into the lungs during IAV infection, and monocytes are susceptible to this infection [40]. Monocyte-derived macrophages in protection from IAV infection have been described [41].

This study had several limitations. First, as a retrospective study, not all clinical information and laboratory data could be collected, and the patients enrolled in this study were limited. Second, all participants in this study were inpatients in our hospital, and no cases of death were included. Thus, selection bias may have occurred. Third, the patient data were not from the same year and therefore a difference in the dose of hematologics was inevitable. Fourth, there was a mismatch in patient age between the groups. Interestingly, a better prognosis in the AdV group can be linked to a medium age, which is fairly lower compared to other groups. Fifth, only patients with COVID-19 were divided by severity, which was not true for the other viral pneumonia groups. Finally, no correlation analysis was performed, and the treatments provided were not specified. Therefore, the leukocytosis may be related to the therapy and not primarily to disease severity.

## Conclusions

Patients with severe COVID-19 had higher neutrophil and lower lymphocyte and HGB counts than those with mild COVID-19 during the disease course. Patients with mild COVID-19 and AdV pneumonia had better outcomes than those with other pneumonia diseases.

One of the COVID-19 characteristics is the continuous decline in HGB count. Neutrophils are related to the aggravation of COVID-19, particularly SARS. Thrombocytopenia was noted in patients with SARS and severe COVID-19 even at the recovery stage, which is consistent with the extreme clinical outcomes. The lymphocyte count was related to the course of AdV, recovery of COVID-19, and disease development of SARS.

## Data Availability

The datasets used and/or analyzed during the current study are available from the corresponding author on reasonable request.

## Abbreviations

COVID-19: Coronavirus disease 2019
SARS: Severe acute respiratory syndrome
HGB: Hemoglobin
PLT: Platelet
WBC: White blood cell
AdV: Adenovirus
IAV: Influenza A virus
H1N1: H1N1 influenza A
MERS: Middle East respiratory syndrome
